# Movement of St. Louis encephalitis virus in the Western United States, 2014- 2018

**DOI:** 10.1371/journal.pntd.0008343

**Published:** 2020-06-10

**Authors:** Daniele M. Swetnam, Jackson B. Stuart, Katherine Young, Payal D. Maharaj, Ying Fang, Sandra Garcia, Christopher M. Barker, Kirk Smith, Marvin S. Godsey, Harry M. Savage, Vonnita Barton, Bethany G. Bolling, Nisha Duggal, Aaron C. Brault, Lark L. Coffey

**Affiliations:** 1 Department of Pathology, Microbiology, and Immunology, School of Veterinary Medicine, University of California, Davis, Davis, California, United States of America; 2 Division of Vector-borne Diseases, Centers for Disease Control, Fort Collins, Colorado, United States of America; 3 Maricopa County Environmental Services Department, Phoenix, Arizona, United States of America; 4 Idaho Bureau of Laboratories, Boise, Idaho, United States of America; 5 Laboratory Services Section, Texas Department of State Health Services, Austin, Texas, United States of America; 6 Department of Molecular Biology, College of Veterinary Medicine, Virginia Polytechnic Institute and State University, Blacksburg, Virginia, United States of America; Lowell General Hospital, UNITED STATES

## Abstract

St. Louis encephalitis virus (SLEV) is a flavivirus that circulates in an enzootic cycle between birds and mosquitoes and can also infect humans to cause febrile disease and sometimes encephalitis. Although SLEV is endemic to the United States, no activity was detected in California during the years 2004 through 2014, despite continuous surveillance in mosquitoes and sentinel chickens. In 2015, SLEV-positive mosquito pools were detected in Maricopa County, Arizona, concurrent with an outbreak of human SLEV disease. SLEV-positive mosquito pools were also detected in southeastern California and Nevada in summer 2015. From 2016 to 2018, SLEV was detected in mosquito pools throughout southern and central California, Oregon, Idaho, and Texas. To understand genetic relatedness and geographic dispersal of SLEV in the western United States since 2015, we sequenced four historical genomes (3 from California and 1 from Louisiana) and 26 contemporary SLEV genomes from mosquito pools from locations across the western US. Bayesian phylogeographic approaches were then applied to map the recent spread of SLEV. Three routes of SLEV dispersal in the western United States were identified: Arizona to southern California, Arizona to Central California, and Arizona to all locations east of the Sierra Nevada mountains. Given the topography of the Western United States, these routes may have been limited by mountain ranges that influence the movement of avian reservoirs and mosquito vectors, which probably represents the primary mechanism of SLEV dispersal. Our analysis detected repeated SLEV introductions from Arizona into southern California and limited evidence of year-to-year persistence of genomes of the same ancestry. By contrast, genetic tracing suggests that all SLEV activity since 2015 in central California is the result of a single persistent SLEV introduction. The identification of natural barriers that influence SLEV dispersal enhances our understanding of arbovirus ecology in the western United States and may also support regional public health agencies in implementing more targeted vector mitigation efforts to protect their communities more effectively.

## Author summary

Following the detection of West Nile virus in the United States, evidence of the historically endemic and closely related virus, St. Louis encephalitis virus (SLEV), dropped nationwide. However, in 2015, a novel genotype of SLEV, previously restricted to Argentina, was identified as the etiological agent of an outbreak of neurological disease in Arizona, United States. Since that time, the genotype has expanded throughout the Western United States, including into California, Nevada, Texas, Idaho, and Oregon. In this study, samples containing SLEV, provided by public health and mosquito abatement agencies, were sequenced and used in phylogenetic analyses to infer patterns of SLEV movement. Three independent routes of SLEV dispersal were identified: Arizona to Southern California, Arizona to Central California, and Arizona to all locations east of the Sierra Nevada mountains. The Sierra Nevada mountains and the Transverse Ranges appear to separate the three routes of SLEV movement, suggesting that geographic features may act as barriers to virus dispersal. Identification of patterns of SLEV dispersal can support regional public health agencies in improving vector mitigation efforts to protect their communities more effectively.

## Introduction

St. Louis encephalitis virus (SLEV) is an arthropod-borne flavivirus (*Flaviviridae*, *Flavivirus*) maintained in an enzootic cycle involving *Culex* spp. mosquitoes and passeriform and columbiform birds. While SLEV infections are non-fatal in birds, spillover into humans [1] and horses [2] can result in significant and sometimes fatal neurological disease. The genome of SLEV is encoded by a single-stranded, positive-sense RNA genome consisting of one open reading frame (ORF) and non-coding regions at the 5’ and 3’ ends. The ORF is translated as a single polyprotein that is co- and post-translationally cleaved into three structural proteins and seven nonstructural (NS) proteins: Capsid (C), Envelope (E), pre-membrane (prM), NS1, NS2A, NS2B, NS3, NS4A, NS4B, and NS5.

St. Louis encephalitis virus occurs throughout North and South America, as well as in the Caribbean islands [3]. Genetic variation of SLEV from different locations has been characterized using oligonucleotide fingerprinting [4], single-strand conformation polymorphism (SSCP) [5], base exclusion sequence scanning [6] and phylogenetic [7–11] methods. The most recent phylogenetic studies have classified SLEV into eight genotypes: I-VII and Palenque [7–11].

In the United States (US), sporadic focal outbreaks have been reported since SLEV was first detected in 1933 [12]. Endemic activity in the absence of outbreaks also has been reported in Florida (FL) [13], Texas (TX) [14] and California (CA) [15–17] from 1933 to 2003 (reviewed in [18]). Following the first detection of West Nile virus (WNV, also a flavivirus) in the Americas in 1999, SLEV activity was significantly reduced throughout the US [1]. In CA, SLEV was not detected after 2003, the year WNV was first detected in CA, until 2015 when SLEV-positive mosquito pools and sentinel chickens were detected in Coachella Valley in Riverside County, CA [18]. The re-emergence of SLEV detected in mosquitoes and sentinel chickens in CA was concurrent with an outbreak of human disease in Maricopa County, Arizona (AZ) [19]. Retrospective analyses of WNV-positive mosquito pools collected in Maricopa County, AZ in 2014 detected SLEV RNA in a pool, revealing that SLEV was present in that area at least one year earlier than initially detected. However, it remains unclear if SLEV was present in AZ prior to 2014 because SLEV was not surveyed in that state during the years leading up to the 2015 outbreak and WNV-negative pools were not saved [18].

Prior to 2014, SLEV genotypes I, II [8] and V [11,13] were reported in the US and genotypes III, IV, VI, and VII were thought to be restricted to South America [8,20]. However, recent sequencing and phylogenetic analyses have demonstrated that SLEV detected in AZ and CA during and after 2014 belong to genotype III, which was previously only reported in Argentina in 1978 and 2005 [18]. The emergence of South American SLEV genotypes in the US, now including genotype III, is consistent with previous studies that have detected periodic introductions of other SLEV genotypes from South America into North America, presumably by migrating birds [7–9].

Since 2015, SLEV has been detected each year throughout the western US, ranging from southern CA, AZ, and southern NV, to the northern Central Valley of CA and southeastern Oregon (OR) [21,22]. However, it is unclear if these detections are the result of re-emergence of previously endemic strains or whether they represent expansion of the more recently introduced genotype III SLEV. Furthermore, if the continued detection of SLEV in the western US is the result of expansion of genotype III, epidemiology and mosquito surveillance alone are not sufficient to decipher the specific routes of arbovirus spread, which may provide important insights into the ecological mechanisms influencing SLEV invasion of the western US. The western US is a geographic region that represents a heterogeneous landscape including mountains, coasts, deserts, temperate rainforests, urban cities, and farmlands, which could support endemic SLEV transmission cycles that use different hosts, vectors and mechanisms of persistence.

Given that SLEV transmission is dependent on host-vector interactions, ecological features influencing the spatial distribution of susceptible bird and mosquito species are likely to impact SLEV spread. Seroprevalence and experimental infection data show that house finches, house sparrows, common ground-doves [23] and nestling mourning doves [24] are the most important SLEV amplifying hosts in CA. House finches [25], house sparrows [26], and common ground doves [27] are resident birds that migrate short distances, while mourning doves [28] undertake long-distance migrations each year. However, banding studies that involve the capture, marking, and recapture of individual birds have demonstrated the range and overall migration distance of individual birds is highly variable even among resident birds, which have been detected more than 1000 km from their initial capture location [29].

The most important vectors for SLEV are *Culex tarsalis* [30,31] and several species in the *Cx*. *pipiens* complex, including *Cx*. *pipiens* and *Cx*. *quinquefasciatus* [31]. Typically, dispersal of *Cx*. *pipiens* [32], *Cx*. *quinquefasciatus* [33] and *Cx*. *tarsalis* is limited to distances of < 3 km; however longer-range dispersal has been reported on several occasions among female *Cx*. *tarsalis* mosquitoes in CA, including in the Coachella Valley of southeastern CA (5.7 km) [32] and the southern Central Valley of CA (12.6 km) [34]. Humans can also facilitate long-distance movement of mosquitoes by transport in vehicles [35].

**Fig 1:**
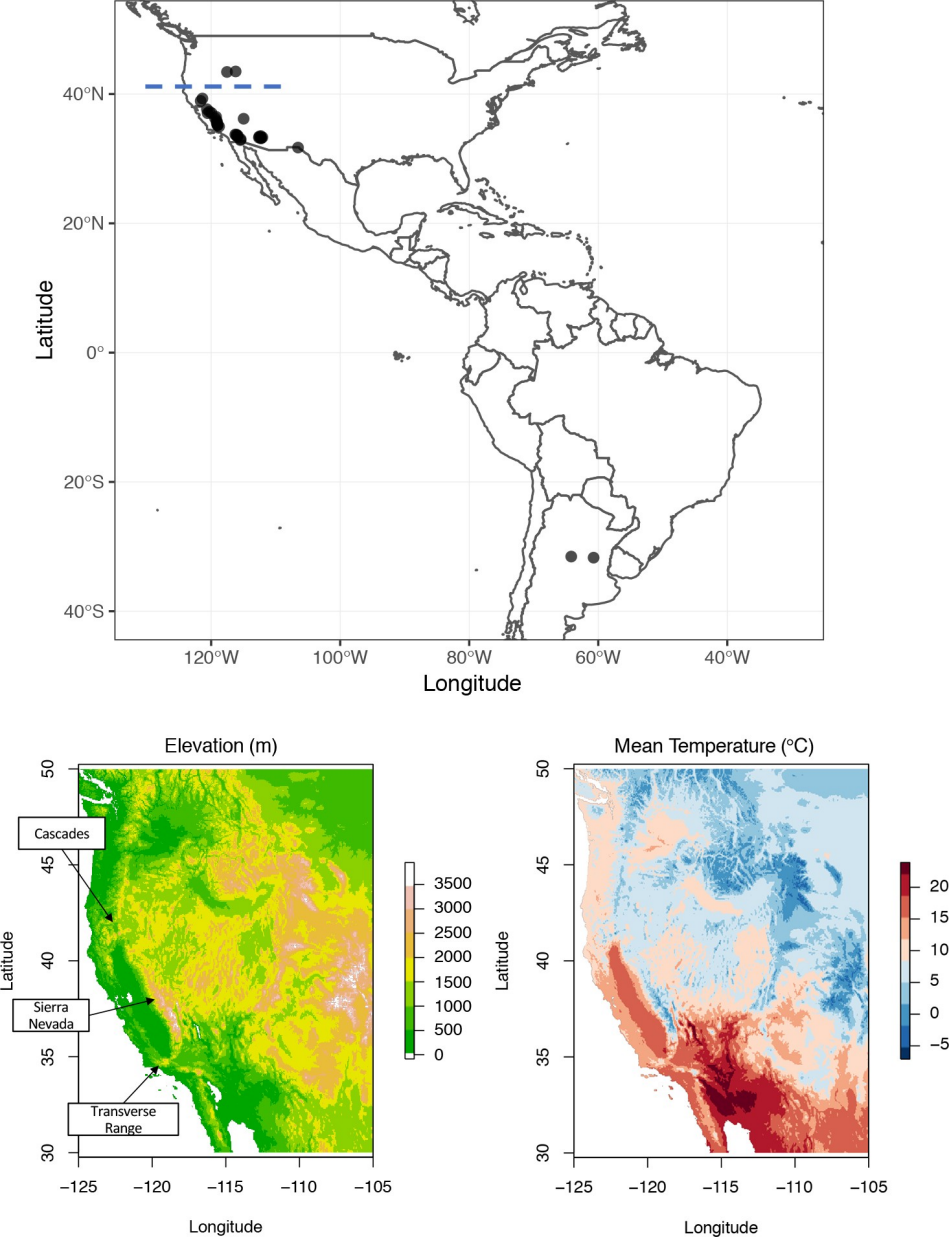
(A): **Map of the Americas showing the distribution of genotype III St. Louis encephalitis virus genomes used in this study (circles).** The 40°N latitude line is shown by a blue hatched line. The varied landscape of the Western US is shown, including (B) elevation with several mountain ranges highlighted and annual mean temperature (C).

The goal of this study was to understand SLEV movement within CA and the broader western US. We generated full ORF sequences of 4 historic SLEV isolates and 26 SLEV-positive mosquito pools collected in ecologically distinct regions of AZ, CA, NV, OR and Idaho (ID) (Fig 1) and then characterized their genetic relatedness and patterns of spread using Bayesian phylogeographic approaches. Our results show that all SLEV genomes detected in the western US since 2015 belong to genotype III, providing no evidence that any historically endemic non-genotype III SLEV continue to circulate in the western US. Within genotype III, three distinct routes of SLEV movement were detected. The routes appear to have been influenced by three mountain ranges in the western US that likely restrict the movement of SLEV mosquito vectors and avian reservoirs. The identification of these natural barriers enhances our understanding of arbovirus ecology in the western US and may also support regional public health agencies in implementing more effective strategies for protecting their communities. For example, augmenting vector mitigation efforts in low elevation valleys, where natural barriers are more likely to be permissive to virus expansion, could prevent virus transmission into new areas. These findings also highlight the importance of collaboration between academic institutions and local public health programs in the pursuit of a more thorough understanding of infectious disease circulation.

## Materials and methods

### Mosquito pool sources

Mosquito pools collected in CA were procured through routine arbovirus surveillance conducted by local mosquito abatement districts and the Davis Arbovirus Research and Training laboratory at the University of California, Davis. Additional SLEV-positive mosquito pools were provided by the Texas Department of State Health Services, the Idaho Department of Health & Welfare, the Oregon Veterinary Diagnostic Laboratory, and the Southern Nevada Health District. A full list of the SLEV-positive samples, and the locations and dates of collection included in this study is shown in Table 1.

**Table 1:** SLEV sequences and metadata.

Accession	Strain	Date	Location	Source	Genotype	Collected by
AY632544	Argentina 66	1966	Córdoba, Argentina	*Calomys musculinus*	VII	NA
DQ359217	MSI 7	1975	Mississippi, US	Unknown	II	NA
EF158048	BE AR 23379	1960	Para, Brazil	*Sabethes belisarioi*	V	NA
EF158049	904.3	1955	Kentucky, US	*Colaptes auratus*	II	NA
EF158050	MSI 7	1975	Mississippi, US	*Passer domesticus*	II	NA
EF158051	GMO 94	1969	Guatemala	*Culex nigripalpus*	II	NA
EF158052	V 2380–42	2001	Texas, US	*Culex quinquefasciatus*	II	NA
EF158053	BeAn 246262	1973	Para, Brazil	*Didelphis marsupialis*	V	NA
EF158054	75 D 90	1975	Peru	Unknown	V	NA
EF158055	TBH 28	1962	Florida, US	*Homo sapiens*	II	NA
EF158056	TRVL 9464	1955	Trinidad	*Psorophora ferox*	V	NA
EF158057	78 A 28	1978	Guatemala	Unknown	II	NA
EF158058	Kern 217	1989	Kern County, CA, US	*Culex tarsalis*	II	NA
EF158059	65 V 310	1961	Mexico	Unknown	II	NA
EF158060	GML 903797	1983	Panama	Unknown	VI	NA
EF158061	69 M 1143	1969	Florida	*Procyon lotor*	II	NA
EF158062	FL 79–411	1979	Florida	*Culex nigripalpus*	II	NA
EF158063	COR AN 9124	1966	Córdoba, Argentina	*Calomys musculinus*	VII	NA
EF158064	GML 902612	1973	Panama	*Haemagogus equinus*	IV	NA
EF158065	TNM 4–711 K	1974	Tennessee, US	*Culex pipiens*	II	NA
EF158066	GHA-3	1955	Haiti	*Butorides virescens*	II	NA
EF158067	BE AN 247377	1973	Para, Brazil	*Hylophilax poecilonota*	V	NA
EF158068	COR AN 9275	1967	Córdoba, Argentina	*Mus musculus*	VII	NA
EF158069	72 V 4749	1972	Colorado, US	*Culex tarsalis*	I	NA
EF158070	Parton	1933	Missouri, US	*Homo sapiens*	II	NA
EU566860	Hubbard	1937	Missouri, US	*Homo sapiens* (brain)	II	NA
FJ753286	CbaAR-4005	2/15/05	Córdoba, Argentina	*Culex quinquefasciatus*	III	NA
FJ753287	79V-2533	1978	Santa Fe, Argentina	*Culex* spp.	III	NA
JF460774	IMP115	2003	CA, US	*Culex tarsalis*	V	NA
JQ957868	Palenque-C475	2008	Mexico	*Culex nigripalpus*	PAL	NA
JQ957869	Palenque-A770	2008	Mexico	*Culex nigripalpus*	PAL	NA
KF589299	FLU3632	3/27/06	Peru	*Homo sapiens* (oropharyngeal swab)	V	NA
KM267635	BeH355964	1978	Para, Brazil	*Homo sapiens*	V	NA
KT823415	RT121B	7/7/15	Maricopa County, AZ	*Culex quinquefasciatus*	III	NA
KX258460	AZ43	7/14/15	Maricopa County, AZ, US	*Culex tarsalis*	III	NA
KX258461	COAV2281	7/28/15	Coachella Valley, CA, US	*Culex tarsalis*	III	NA
KX258462	AZ39	7/14/15	Maricopa County, AZ, US	*Culex quinquefasciatus*	III	NA
KX965720	AZ14	2014	AZ, US	*Culex* spp.	III	NA
KY825742	KERN2	2016	Kern County, CA, US	*Culex pipiens*	III	NA
KY825743	KERN1	9/9/16	Kern County, CA, US	*Homo sapiens*	III	NA
MN233306	RT280*	6/15/17	Maricopa County, AZ, US	*Culex tarsalis*	III	Maricopa County Environmental Services Department
MN233307	BUCO327*	8/28/17	Butte County, CA, US	*Culex tarsalis*	III	Butte County Mosquito and Vector Control District
MN233308	COAV3064*	7/26/17	Coachella Valley, CA, US	*Culex tarsalis*	III	Coachella Valley Mosquito and Vector Control District
MN233309	DLNO229*	9/15/17	Delano County, CA, US	*Culex quinquefasciatus*	III	Delano Mosquito Abatement District
MN233310	FRWS650*	10/12/17	Fresno Westside, CA, US	*Culex tarsalis*	III	Fresno Westside Mosquito Abatement District
MN233311	ID17*	9/12/17	Gem County, ID, US	*Culex tarsalis*	III	Idaho Bureau of Laboratories
MN233312	IMPR165*	7/20/18	Imperial County, CA, US	*Culex quinquefasciatus*	III	Imperial County Vector Control
MN233313	IMPR570*	9/11/17	Imperial County, CA, US	*Culex tarsalis*	III	Imperial County Vector Control
MN233314	KERN245*	7/5/18	Kern County, CA, US	*Culex quinquefasciatus*	III	Kern Mosquito and Vector Control District
MN233315	KERN345*	7/15/16	Kern County, CA, US	*Culex quinquefasciatus*	III	Kern Mosquito and Vector Control District
MN233316	KERN351*	6/21/17	Kern County, CA, US	*Culex quinquefasciatus*	III	Kern Mosquito and Vector Control District
MN233317	MADR393*	9/29/17	Madera County, CA, US	*Culex quinquefasciatus*	III	Madera County Mosquito and Vector Control District
MN233318	MERC342*	9/14/17	Merced County, CA, US	*Culex tarsalis*	III	Merced County Mosquito Abatement District
MN233319	NV16*	5/15/16	Clark County, NV, US	*Culex tarsalis*	III	Southern Nevada Health District, Environmental Health Public Accommodations & Mosquito Disease Surveillance
MN233320	OR17*	2017	Malheur County, OR, US	*Culex* spp.	III	Oregon Veterinary Diagnostic Laboratory
MN233321	SUYA288*	7/31/17	Sutter/Yuba County, CA, US	*Culex tarsalis*	III	Sutter-Yuba Mosquito and Vector Control District
MN233322	TLRE179*	8/16/17	Tulare, CA, US	*Culex quinquefasciatus*	III	Tulare Mosquito Abatement District
MN233323	TRLK660*	8/3/17	Turlock, CA, US	*Culex pipiens*	III	Turlock Mosquito Abatement District
MN233324	AR15-6004*	7/21/2015	El Paso, TX, US	*Culex quinquefasciatus*	III	Texas Department of State Health Services, Arbovirus-Entomology Laboratory
MN233325	WEST13*	7/20/16	Kern County, CA, US	*Culex tarsalis*	III	West Side Mosquito and Vector Control District
MN233326	COAV2623*	8/25/15	Coachella Valley, CA, US	*Culex tarsalis*	III	Coachella Valley Mosquito and Vector Control District
MN233327	COAV2361*	8/4/15	Coachella Valley, CA, US	*Culex tarsalis*	III	Coachella Valley Mosquito and Vector Control District
MN233328	COAV2616*	8/25/15	Coachella Valley, CA, US	*Culex tarsalis*	III	Coachella Valley Mosquito and Vector Control District
MN233329	RT496*	7/10/15	Maricopa County, AZ, US	*Culex quinquefasciatus*	III	Maricopa County Environmental Services Department
MN233330	RT246*	7/21/15	Maricopa County, AZ, US	*Culex quinquefasciatus*	III	Maricopa County Environmental Services Department
MN233331	LA-01-4278*	8/30/2001	Ouachita Parish, LA, US	*Culex quinquefasciatus*	II	Center for Disease Control
MN233332	BFS1750*	1953	Kern County, CA, US	*Culex tarsalis*	I	Kern Mosquito and Vector Control District
MN233333	COAV750*	1998	Coachella Valley, CA, US	*Culex tarsalis*	I	Coachella Valley Mosquito and Vector Control District
MN233334	KERN217*	1989	Kern County, CA, US	*Culex tarsalis*	II	Kern Mosquito and Vector Control District
MN233335	TLRE15*	6/20/18	Tulare, CA, US	*Culex quinquefasciatus*	III	Maricopa County Environmental Services Department

The accession number and strain name of each sequence used in this study are summarized with the associated genotype and metadata including location and date. Where possible, the full date including month and day was provided (mm/dd/yyyy). Novel genomes generated in this study are indicated with an asterisk (*).

### SLEV RNA extraction and genome sequencing from mosquito pools

SLEV-positive mosquito pools from CA were identified using triplex reverse transcription polymerase chain reaction (RT-PCR) [36]. Mosquito pools from other states were identified as SLEV-positive through similar molecular diagnostic approaches. Mosquito pools were homogenized in 1–2 mL of virus transport medium (VTM, which was 10% fetal bovine serum [FBS], 50 ug/mL gentamicin, 1% penicillin/streptomycin, and 5 ug/mL amphotericin B) for 2 minutes and clarified by centrifugation. Homogenized mosquito pools were filtered with a 0.45 μm syringe filter. SLEV RNA was then extracted from 140 μl of each mosquito pool filtrate using a QIAamp® Viral RNA Mini Kit (Qiagen, Hilden) in accordance with the manufacturer’s recommendations. SLEV RNA was eluted into 40 μl of nuclease free water. The extracted SLEV RNA was then amplified for sequencing using a Qiagen® OneStep Reverse transcription (RT)-Polymerase chain reaction (PCR) kit (Qiagen, Hilden). Each reaction contained 14 μl nuclease free water, 5 μl 5x RT-PCR Buffer, 1 μl dNTP Mix (10 mM of each dNTP), 1 μl RT-PCR Enzyme Mix (Omniscript Reverse Transcriptase, Sensiscript Reverse Transcriptase, and HotStarTaq® DNA Polymerase), 0.5 μl each of ‘forward’ and ‘reverse’ primers (Table 2), and 3 μl extracted SLEV RNA. The thermal cycler conditions were as follows: a 30-minute reverse transcription step at 50°C, a 15-minute initial PCR activation step at 95°C, 40 cycles of a 3-step cycling phase (including a 1-minute denaturation step at 94°C, a 1-minute annealing step at 57°C, and a 2-minute extension step at 72°C), and a 10-minute final extension step at 72°C. All reactions were then held at 4°C.

**Table 2:** Primers used to amplify and sequence St. Louis encephalitis virus.

Forward primer name	Forward primer sequence	Reverse primer name	Reverse primer sequence
F1	GAGCGGAGAGGAAACAGATTT	800R	AAAGAGATGTTGTGGACCGT
636p	GCATGGGACATTCAAGGCG	1963n	GACCGTGACCAATCTTCCAA
1874p	TACACTGGAAGCAACGGACC	3145n	TTTAGGGCCGCCTAGTGTTA
3068p	CCAGAAACGCACACCCTATG	3784n	AGCTGCTCCAATAACCATCA
3689p	GCTGTCTTCAAAGTGCAACC	4999n	ACCCTGTCCAATCAGTACCC
4893p	GAGCCGTGACTCTTGATTTCC	6182n	CGTTGGAGGCCACTTTGTAAG
5958p	ATGAGGACGACCACGATTTG	7216n	GCATTTATGATCCCAGGATGG
7125p	TGCTGGGGTGTTGGAATCAA	8386n	ATGTGAATTTGGGAAGTGGAACG
8314p	CATGGGAAGGATGGACAAACAG	9009n	GGAGAATTTGGGAAGGCTAAAGG
9000F	CCAAAGTTCTGGGAAATGGTT	R6	ATTTCACCAGGAGCAGGATG
F7	GGTTGAGTGGCTAAGGAAGAA	R14	TAAACGGTGCTGTCTGTAACC

Each row represents a primer pair.

SLEV RT-PCR products were subjected to electrophoresis on 1% agarose gels and purified according to the manufacturer’s recommendations using a QIAquick® PCR Purification Kit (Qiagen, Hilden) or a QIAquick® Gel Extraction Kit (Qiagen, Hilden). Purified complementary DNA was eluted into 40 μl of Buffer EB (Qiagen, Hilden) and Sanger sequenced using the primers in Table 2 at the DNA Sequencing Core facilities at UC Davis or the Division of Vector-Borne Diseases at the Centers for Disease Control and Prevention (DVBD-CDC). The resulting sequences were aligned using a published reference sequence (GenBank accession number: KX258462) to generate a consensus sequence using Sequencher® DNA Sequence Analysis Software (Gene Codes Corporation, Ann Arbor). Prior to alignment, the sequences were trimmed using the Sequencher command “Trim Ends” in accordance with the software’s suggested trim criteria. An average of double coverage at each coding genomic position was achieved, and sequences were called by verifying that the chromatogram peaks were both clear and consistent across all strands at each nucleotide position.

### Phylogenetic analyses

Consensus genomic SLEV sequences were aligned with all previously published SLEV genomes available in GenBank (Table 1) in Mega7.0.26 [37]. Only genomes that contained at least 99% of the SLEV ORF were included in the alignment. A nucleotide substitution model was identified by comparing 88 models using Akaike and Bayesian information criterion in jModelTest2 [38] on a CIPRES Science Gateway [39]. The evolutionary history was inferred by using a Maximum Likelihood method in Mega7.0.26 [37]. The tree with the highest log-likelihood of 500 bootstraps is shown. The tree is drawn to scale, with branch lengths measured in the number of substitutions per site. All positions containing gaps and missing data were eliminated. The tree was visualized in FigTree v1.4.3 and rooted using the midpoint root function.

### Phylogeographic analyses

Bayesian phylogeographic approaches were used to investigate the spatial expansion of SLEV since 2015. Since Bayesian phylogenetic methods incorporate time into their reconstructions, the alignment was first evaluated to determine the strength of the temporal signal. The temporal signal was evaluated by comparing the collection date of each mosquito pool with the phylogenetic distance (root-to-tip distance) in the Maximum Likelihood tree in TempEST [40]. The relationship between collection date and root-to-tip distance of each genome was compared using linear regression and a Pearson correlation test in R version 3.5.3 [41].

Genomes belonging to SLEV genotype III were used to further investigate the geospatial diffusion patterns of SLEV in the western US using a Bayesian phylogeographic platform implemented through BEAST v1.10.4 [42]. Two partial SLEV genomes from AZ, AZ14 (GenBank accession number: KX965720) and RT246 (GenBank accession number: MN233330), for which approximately 85% and 90% of the genome, respectively, were available, were also included to maximize use of all available SLEV genomic information.

Standard path-sampling and stepping-stone approaches were used to determine the optimal combination of clock model (fixed molecular clock), tree prior (Bayesian skyline) and continuous trait diffusion model (Cauchy). Each Markov Chain Monte Carlo (MCMC) process was sampled for 50 million steps, and every 5,000^th^ step was recorded. Log files were inspected visually in Tracer to confirm that each prior underwent adequate mixing and the MCMC chain achieved topological convergence. Three independent MCMC chains were combined in LogCombiner and 10% burn-in was removed. Maximum clade credibility trees were annotated in TreeAnnotator. Both LogCombiner and TreeAnnotator are available through the BEAST v1.10.4 package [42]. Phylogenies were visualized in FigTree and geospatial reconstruction was performed in SpreaD3 [43].

### Map preparation and statistical testing

Mapping and statistical testing were performed in R version 3.5.3 [41]. Maps were generated using the following packages: dismo, ggplot, rasrer, rnaturalearth, rnaturalearthdata and sf. Elevation and temperature data were data provided by WorldClim version 2 (resolution 2.5 minutes) and represent means for years 1970–2000 [44].

## Results

### Genome sequencing of SLEV from the Western United States, 2015–2018

The full ORF sequences were determined for 26 SLEV-positive mosquito pools from 3 species collected from 2015–2018 in CA (n = 19), TX (n = 1), NV (n = 1), AZ (n = 3), OR (n = 1) and, ID (n = 1) (Table 1). Additional ORF sequences from four historical SLEV strains were also determined and included: BFS1750 (Kern County, CA 1953), COAV750 (Coachella Valley, CA 1988), KERN217 (Kern County, CA 1989), and LA-01-4278 (Monroe, Louisiana 2001). The consensus sequences generated in this study were aligned with 40 previously published SLEV genomes and were deposited in GenBank (GenBank accession numbers: MN233306-MN233335).

### Phylogenetic analyses

**Fig 2:**
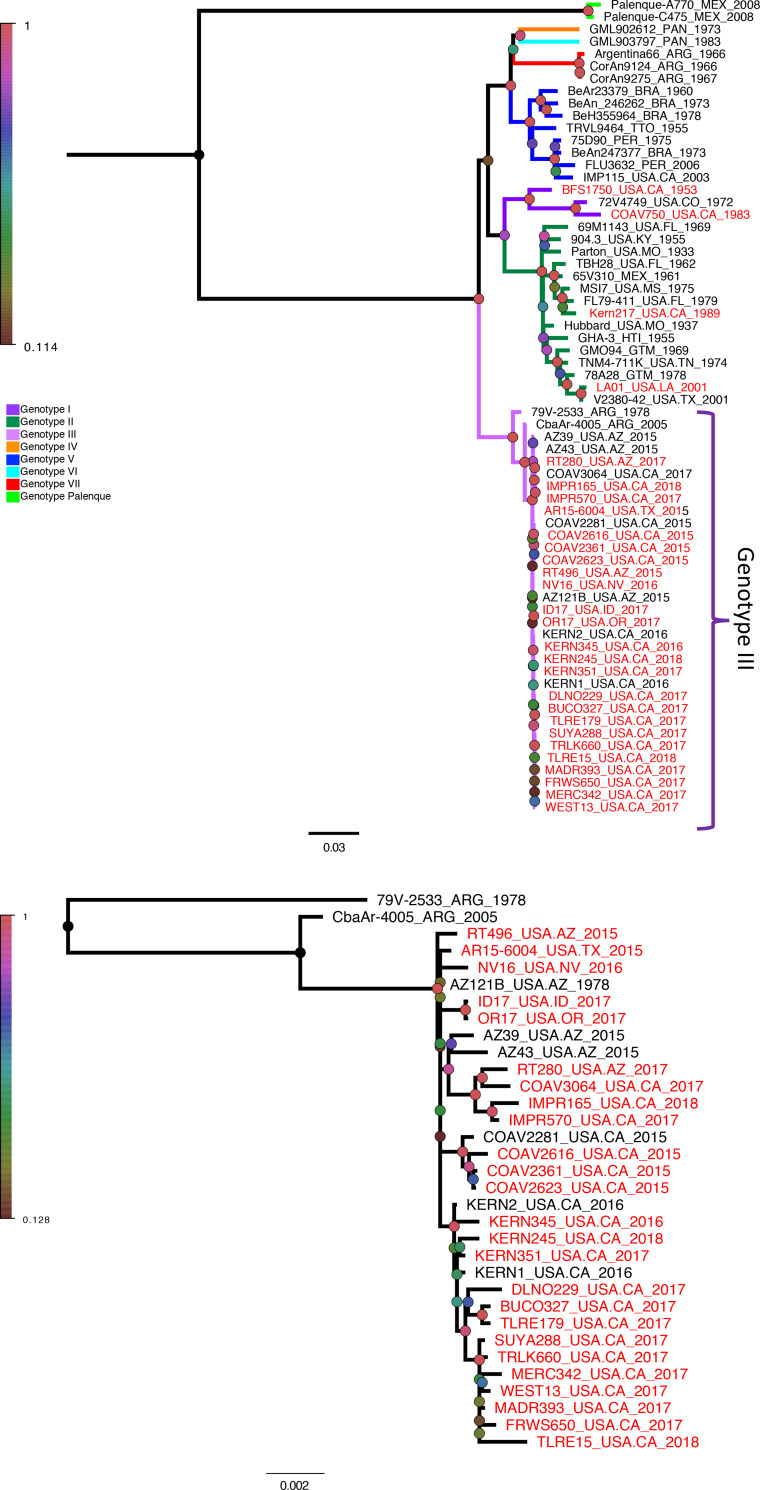
Maximum Likelihood phylogenetic tree highlighting the evolutionary history of SLEV genotype III in the Western US. SLEV genomes comprising all genotypes (A) or genotype III only (B) are shown. Genomes in red were sequenced for this study. For panel A, branch colors denote genotypes represented by the key. For both A and B, the percentage of trees in which the associated genomes clustered together is indicated by the colored circles at nodes with pink indicating high support (1) and brown indicating low support (0). Branch length is scaled to reflect the number of substitutions per site. The branch length scale for both trees is provided under the tree in B. Genomes are named as follows: Strain_Location_Year. Abbreviations: Argentina, ARG; Arizona AZ; Brazil, BRA; California, CA; Colorado, CO; Florida, FL; Guatemala, GTM; Haiti, HTI; Idaho ID; Kentucky, KY; Louisiana, LA; Mexico, MEX; Mississippi, MS; Missouri, MO; Nevada, NV; Oregon, OR; Panama, PAN; Peru, PER; Tennessee, TN; Texas, TX, Trinidad and Tobago, TTO.

The evolutionary history of SLEV genomes was inferred with Maximum Likelihood analyses (Fig 2A). Eight SLEV genotypes were identified and the clustering pattern of all published sequences was consistent with previous reports (Fig 2) [7–11]. All SLEV genomes collected after 2014 clustered together in genotype III, along with two Argentinian sequences (strain name and GenBank accession numbers: CbaAr-4005: FJ753286 and 79V-2533: FJ753287) and six previously published SLEV genomes collected in the Western US (strain name and GenBank accession numbers: AZ14: KX965720, AZ39: KX258462, AZ43: KX258460, COAV2281: KX258461, KERN1: KY825743, KERN2: KY825742). This clustering pattern strongly suggests there was a single introduction of genotype III SLEV from South America into North America, and that all genomes from the western US since 2014 are descendants of a single genotype III ancestor. The historic SLEV genomes clustered in genotypes I (BFS1750 and COAV750) and II (LA01 and KERN217) (Fig 2A). The most appropriate nucleotide substitution model was identified as a general time reversible model with a gamma shape parameter and proportion of invariable sites (GTR + G + I) by Akaike and Bayesian Information Criterion by jModelTest2 (Table 3). The evolutionary history of SLEV genomes was inferred with Maximum Likelihood analyses (Fig 2A).

**Table 3:** Nucleotide Substitution Model Selection.

Model	Negative log-likelihood	BIC	AIC
F81	57977.882	117258.494	116237.764
F81+G	55570.1789	112452.327	111424.358
F81+I	55661.7749	112635.519	111607.55
F81+I+G	55565.9171	112453.043	111417.834
GTR	52851.1233	107051.173	105994.247
GTR+G	50384.2976	102126.76	101062.595
GTR+I	50439.7134	102237.592	101173.427
GTR+I+G	50359.8932	102087.191	101015.786
HKY	53448.9191	108209.807	107181.838
HKY+G	50791.6354	102904.479	101869.271
HKY+I	50905.2909	103131.79	102096.582
HKY+I+G	50781.5802	102893.608	101851.16
JC	58008.1073	117291.227	116292.215
JC+G	55585.8946	112456.041	111449.789
JC+I	55679.6947	112643.641	111637.389
JC+I+G	55581.5697	112456.63	111443.139
K80	53483.8306	108251.913	107245.661
K80+G	50812.464	102918.419	101904.928
K80+I	50930.0426	103153.576	102140.085
K80+I+G	50803.7718	102910.274	101889.544
SYM	52980.1957	107281.6	106246.391
SYM+G	50504.7013	102339.85	101297.403
SYM+I	50566.1488	102462.745	101420.298
SYM+I+G	50485.084	102309.855	101260.168
TIM1	52909.0319	107148.511	106106.064
TIM1+G	50449.015	102237.717	101188.03
TIM1+I	50507.2858	102354.258	101304.572
TIM1+I+G	50427.7324	102204.391	101147.465
TIM1ef	53043.7432	107390.216	106369.486
TIM1ef+G	50572.9905	102457.95	101429.981
TIM1ef+I	50635.1094	102582.188	101554.219
TIM1ef+I+G	50554.9754	102431.159	101395.951
TIM2	52864.7698	107059.987	106017.54
TIM2+G	50414.228	102168.143	101118.456
TIM2+I	50474.6248	102288.936	101239.25
TIM2+I+G	50392.3532	102133.632	101076.706
TIM2ef	52995.1456	107293.021	106272.291
TIM2ef+G	50530.5464	102373.062	101345.093
TIM2ef+I	50595.7329	102503.435	101475.466
TIM2ef+I+G	50512.0088	102345.226	101310.018
TIM3	52914.6032	107159.654	106117.206
TIM3+G	50435.4302	102210.547	101160.86
TIM3+I	50486.883	102313.453	101263.766
TIM3+I+G	50410.8649	102170.656	101113.73
TIM3ef	53048.1007	107398.931	106378.201
TIM3ef+G	50564.0983	102440.166	101412.197
TIM3ef+I	50620.5372	102553.043	101525.074
TIM3ef+I+G	50544.1956	102409.6	101374.391
TPM1	53476.7759	108247.043	107233.552
TPM1+G	50807.4364	102917.603	101896.873
TPM1+I	50925.9805	103154.691	102133.961
TPM1+I+G	50798.7447	102909.459	101881.489
TPM1uf	53441.925	108205.058	107169.85
TPM1uf+G	50787.1936	102904.835	101862.387
TPM1uf+I	50901.7959	103134.039	102091.592
TPM1uf+I+G	50777.1414	102893.97	101844.283
TPM2	53428.3098	108150.11	107136.62
TPM2+G	50766.4113	102835.552	101814.823
TPM2+I	50890.9469	103084.624	102063.894
TPM2+I+G	50757.7144	102827.398	101799.429
TPM2uf	53397.824	108116.856	107081.648
TPM2uf+G	50756.8585	102844.165	101801.717
TPM2uf+I	50875.119	103080.686	102038.238
TPM2uf+I+G	50747.0984	102833.884	101784.197
TPM3	53481.5337	108256.558	107243.067
TPM3+G	50797.4994	102897.729	101876.999
TPM3+I	50912.7576	103128.245	102107.515
TPM3+I+G	50788.2729	102888.515	101860.546
TPM3uf	53448.2772	108217.763	107182.554
TPM3uf+G	50775.4394	102881.326	101838.879
TPM3uf+I	50886.6648	103103.777	102061.33
TPM3uf+I+G	50764.6676	102869.022	101819.335
TrN	52915.8735	107152.955	106117.747
TrN+G	50453.7711	102237.99	101195.542
TrN+I	50511.025	102352.498	101310.05
TrN+I+G	50432.1668	102204.02	101154.334
TrNef	53050.7523	107394.995	106381.505
TrNef+G	50578.1623	102459.054	101438.325
TrNef+I	50639.247	102581.224	101560.494
TrNef+I+G	50559.8385	102431.646	101403.677
TVM	53384.7168	108109.12	107059.434
TVM+G	50729.3802	102807.686	101750.76
TVM+I	50847.0239	103042.974	101986.048
TVM+I+G	50718.848	102795.861	101731.696
TVMef	53414.8055	108141.58	107113.611
TVMef+G	50739.9071	102801.022	101765.814
TVMef+I	50862.8135	103046.835	102011.627
TVMef+I+G	50730.6555	102791.759	101749.311

Summary of all models compared using Bayesian and Akaike Information Criterion.

Given that Maximum Likelihood phylogenies are naive to time, the root-to-tip distance of each sequence was compared with the collection date of each genome to measure the strength of the temporal signal within the phylogeny and to determine if time-aware methods, as applied in Bayesian phylogenetic approaches, are appropriate. Unfortunately, the temporal signal was insufficient to allow further analysis using Bayesian methods (Correlation coefficient = -0.36, p-value = 0.0031). However, the temporal strength was sufficiently strong (Correlation coefficient = 0.99, p-value < 2.2e-16) when the phylogeny only contained sequences belonging to genotype III (Fig 2B).

### Phylogeographic analyses

**Fig 3:**
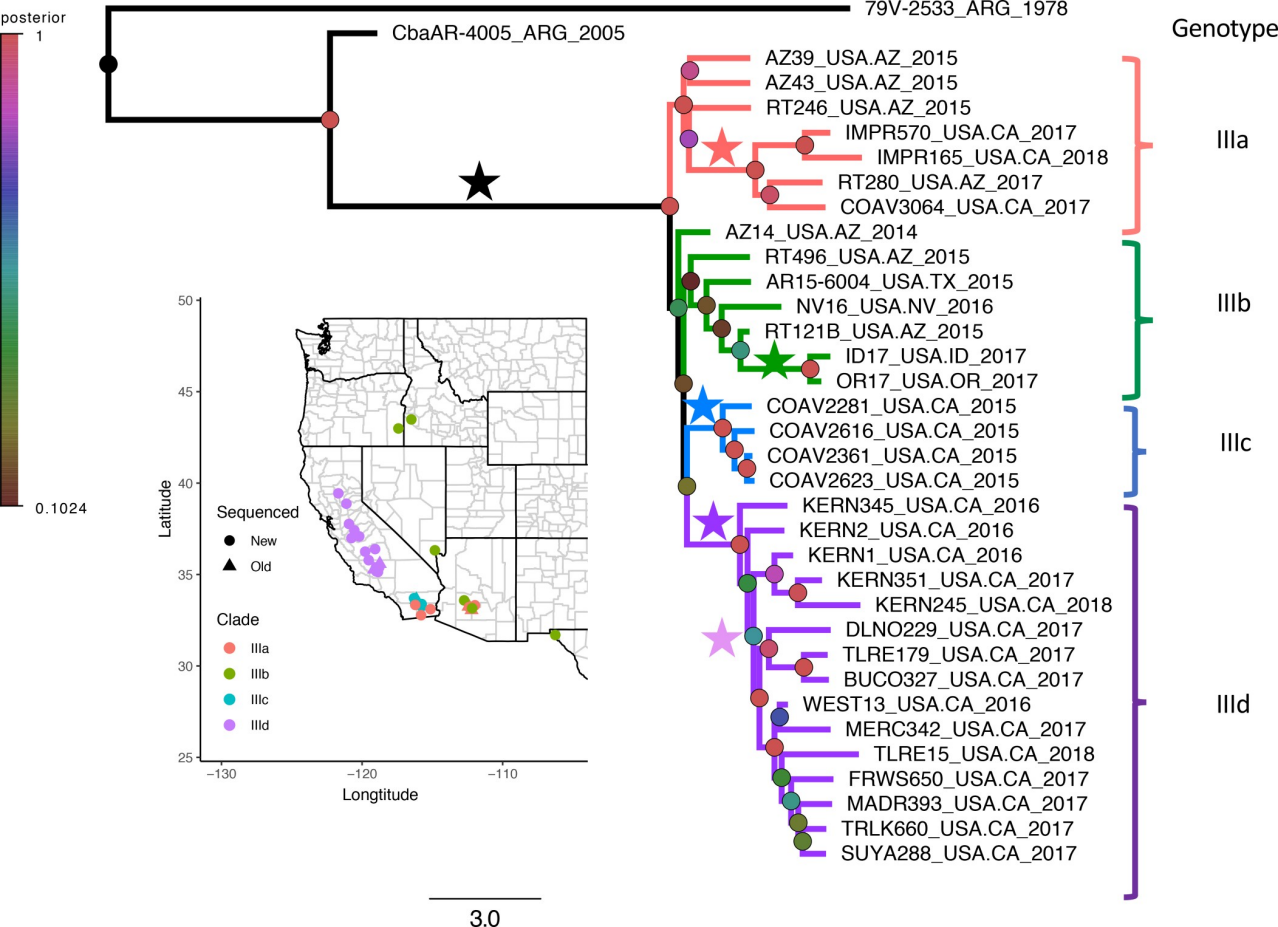
Bayesian phylogeographic analysis showing four distinct clusters of SLEV genotype III in the western US. The branch length is scaled to time in years and the posterior support of each node is represented by the colored circle. Branch length scale is provided at the base of the phylogeny. Colored stars to the left of clades denote shared amino acid substitutions: Black = A-261-V, G-2195-S, L-2210-S, and A-2383-V; Red = G-2288-E; Green = H1079-I and I-2233-V; Blue = I-3095-V; Dark purple = P-2298-S and Light purple = Q-538-R. The map indicates the location of mosquito pool collections. Circles represent new genomes that were generated during this study and triangles represent old genomes sourced from GenBank. Genome names are as follows: Strain_Location_Year. Abbreviations: Argentina, ARG; Arizona, AZ; California, CA; Idaho, ID; Nevada, NV; Oregon, OR; Texas, TX.

The evolutionary histories of the genotype III SLEV sequences were further investigated using a Bayesian approach which incorporates sampling time into reconstructions. The SLEV genomes after 2014 clustered into four groups which we are identifying as IIIa-d (Fig 3). The clustering pattern of the Bayesian phylogeny supported the results obtained using Maximum Likelihood methods. Greater resolution was achieved using the Bayesian model, which is not surprising as the method allows for the inclusion of priors and selects heavily against polytomies. The most recent common ancestor occurred in approximately March of 2013 (95% highest posterior density (HPD) 2012.7 and 2013.8). Clusters IIIa and IIIc originated in AZ and spread into southern CA while remaining south of the Transverse Ranges that form the southern boundary of the Central Valley. Cluster IIIa (posterior = 1) comprises genomes from the southwestern US, including AZ and southern CA from 2015 until 2018, three genomes from AZ in 2015, one from AZ in 2017, and three from southern CA in 2017–2018. Cluster IIIc (posterior = 1) appeared to be geographically restricted, only containing genomes from mosquito pools from Coachella Valley, Riverside County, in southern CA during 2015. Cluster IIIb (posterior = 0.2) contained three genomes from AZ from 2014–2015, as well as all genomes from east of the Sierra Nevada mountains (NV 2016, TX 2015, ID 2017, and OR 2017). However, given the poor support of cluster IIIb, it is unclear how the genomes in this cluster are related to each other. Finally, cluster IIId (posterior = 1) contained 14 genomes from 2016 and 2018 in the Central Valley of CA, which is surrounded by mountain ranges.

Sixty-three amino acid differences were identified among all of the North American SLEV isolates compared to the 2005 genotype III strain from Argentina, CbaAR-4005 (Table 4). All North American genotype III SLEV genomes differed from CbaAR-4005 by three amino acid substitutions: prM V-140-A, NS2A G-79-S, and NS5 I-107-V, and all but one genome (RT246) contained the amino acid substitution NS4A L-18-S. Four genomes within cluster IIIa (AZ17, COAV3064, IMPR165 and IMPR570) shared the amino acid change NS4A G-96-E. Two genomes within cluster IIIb: ID17 and OR17, shared amino acid substitution NS1 H-216-Y and NS4A I-41-V. All genomes in cluster IIIc shared amino acid substitution NS5 I-496-V. All genomes within cluster IIId contained an amino acid substitution NS4A P-106-S and seven of the genomes within cluster IIId contained a second amino acid substitution, E Q-175-R. While it is possible that some or all of these amino acid substitutions arose from stochastic variation, additional studies are needed to determine if they confer a change in infectivity or transmissibility of SLEV in the western US.

**Table 4:** Summary of amino acid substitutions between SLEV strain CbaAr-4005 (GenBank accession number FJ753286) and the SLEV genomes sequenced in the present study.

AZ43	AZ39	RT246	AZ17	COAV3064	IMPR165	IMPR570	COAV2281	COAV2616	COAV2361	COAV2623	RT496	AZ14	NV16	TX15	RT121B	ID17	OR17	KERN345	KERN1	KERN2	KERN245	KERN351	BUCO327	DLNO229	FRWS650	MADR393	MERC342	SUYA288	TRLK660	TLRE15	WEST13	CbaAR-4005	Polyprotein	Position	Protein
.	.	*	.	.	V	V	.	.	.	.	.	.	.	.	.	.	.	.	.	.	.	.	.	.	.	.	.	.	.	.	.	A	71	71	C
.	.	*	.	.	.	.	.	.	.	.	.	.	.	.	.	.	.	M	.	.	.	.	.	.	.	.	.	.	.	.	.	L	92	93	C
.	.	*	.	.	.	.	.	.	.	.	.	.	.	.	.	.	.	.	.	.	.	.	.	.	.	.	.	.	.	I	.	T	154	33	prM
.	A	*	.	.	.	.	.	.	.	.	.	.	.	.	.	.	.	.	.	.	.	.	.	.	.	.	.	.	.	.	.	V	195	74	prM
V	V	*	V	V	V	V	V	V	V	V	V	V	V	V	V	V	V	V	V	V	V	V	V	V	V	V	V	V	V	V	V	A	261	140	prM
.	.	.	.	N	.	.	.	.	.	.	.	.	.	.	.	.	.	.	.	.	.	.	.	.	.	.	.	.	.	.	.	T	365	2	E
.	.	.	S	.	.	.	.	.	.	.	.	.	.	.	.	.	.	.	.	.	.	.	.	.	.	.	.	.	.	.	.	N	450	87	E
.	.	.	.	.	.	.	.	.	.	I	.	.	.	.	.	.	.	.	.	.	.	.	.	.	.	.	.	.	.	.	.	T	493	130	E
.	.	.	.	.	.	.	.	.	.	.	.	.	.	.	.	.	.	.	.	.	.	.	.	.	R	R	R	R	R	R	R	I	538	175	E
.	.	.	.	.	.	.	.	.	.	.	.	.	.	.	.	.	.	.	.	.	.	.	.	.	.	.	.	.	.	Y	.	H	573	210	E
.	.	.	.	.	.	.	.	.	.	.	.	.	.	.	.	.	.	.	.	.	.	.	.	.	.	.	.	.	.	M	.	V	613	250	E
.	.	.	.	.	.	.	.	.	.	.	.	.	.	.	.	.	.	N	.	.	.	.	.	.	.	.	.	.	.	.	.	D	796	433	E
.	.	.	.	.	.	.	.	.	.	.	.	.	.	.	.	.	.	.	L	.	.	.	.	.	.	.	.	.	.	.	.	F	824	461	E
.	.	.	.	.	.	.	.	.	.	.	.	Y	.	.	.	.	.	.	.	.	.	.	.	.	.	.	.	.	.	.	.	H	839	476	E
.	.	.	.	.	.	.	.	.	.	.	.	.	.	.	.	Y	Y	.	.	.	.	.	.	.	.	.	.	.	.	.	.	H	1079	216	NS1
.	.	.	.	.	.	.	.	.	.	.	.	.	.	.	.	.	.	.	.	.	.	.	.	.	.	.	.	.	.	A	.	V	1143	280	NS1
.	.	.	.	.	L	.	.	.	.	.	.	.	.	.	.	.	.	.	.	.	.	.	.	.	.	.	.	.	.	.	.	F	1150	287	NS1
.	.	.	C	.	.	.	.	.	.	.	.	.	.	.	.	.	.	.	.	.	.	.	.	.	.	.	.	.	.	.	.	R	1167	304	NS1
.	.	.	.	.	.	.	.	.	.	.	D	.	.	.	.	.	.	.	.	.	.	.	.	.	.	.	.	.	.	.	.	N	1189	326	NS1
A	.	.	.	.	.	.	.	.	.	.	.	.	.	.	.	.	.	.	.	.	.	.	.	.	.	.	.	.	.	.	.	V	1202	339	NS1
.	.	.	.	.	.	.	.	.	.	.	.	.	.	.	.	.	.	.	.	.	.	.	.	.	.	.	.	.	.	S	.	A	1219	3	NS2A
.	.	.	.	.	.	.	.	.	.	.	R	.	.	.	.	.	.	.	.	.	.	.	.	.	.	.	.	.	.	.	.	Q	1239	23	NS2A
.	.	.	.	.	.	.	.	.	.	.	.	.	.	.	.	.	.	V	.	.	.	.	.	.	.	.	.	.	.	.	.	I	1250	34	NS2A
.	.	I	.	.	.	.	.	.	.	.	.	.	.	.	.	.	.	.	.	.	.	.	.	.	.	.	.	.	.	.	.	L	1267	51	NS2A
.	T	.	.	.	.	.	.	.	.	.	.	.	.	.	.	.	.	.	.	.	.	.	.	.	.	.	.	.	.	.	.	M	1283	67	NS2A
.	.	.	F	.	.	.	.	.	.	.	.	V	.	.	.	.	.	.	.	.	.	.	.	.	.	.	.	.	.	.	.	I	1295	79	NS2A
.	.	.	.	.	.	.	.	.	.	.	.	S	.	.	.	.	.	.	.	.	.	.	.	.	.	.	.	.	.	.	.	C	1297	81	NS2A
.	.	.	.	.	.	.	.	.	.	.	.	I	.	.	.	.	.	.	.	.	.	.	.	.	.	.	.	.	.	.	.	R	1312	96	NS2A
.	.	.	V	.	.	.	.	.	.	.	.	.	.	.	.	.	.	.	.	.	.	.	.	.	.	.	.	.	.	.	.	A	1347	131	NS2A
.	.	S	.	.	.	.	.	.	.	.	.	.	.	.	.	.	.	.	.	.	.	.	.	.	.	.	.	.	.	.	.	L	1389	173	NS2A
.	.	T	.	.	.	.	.	.	.	.	.	.	.	.	.	.	.	.	.	.	.	.	.	.	.	.	.	.	.	.	.	M	1397	181	NS2A
.	.	H	.	.	.	.	.	.	.	.	.	.	.	.	.	.	.	.	.	.	.	.	.	.	.	.	.	.	.	.	.	Y	1408	192	NS2A
.	.	.	G	.	.	.	.	.	.	.	.	.	.	.	.	.	.	.	.	.	.	.	.	.	.	.	.	.	.	.	.	D	1458	16	NS2B
.	.	.	.	.	.	.	.	.	.	.	.	.	.	.	.	.	.	.	.	.	.	.	H	.	.	.	.	.	.	.	.	P	1459	17	NS2B
.	.	.	.	.	.	.	.	.	.	.	.	.	.	.	.	.	.	.	.	.	.	.	.	S	.	.	.	.	.	.	.	P	1604	30	NS3
.	.	.	.	.	.	.	.	.	.	.	.	.	Y	.	.	.	.	.	.	.	.	.	.	.	.	.	.	.	.	.	.	H	1694	120	NS3
.	.	.	.	.	.	.	.	.	.	.	H	.	.	.	.	.	.	.	.	.	.	.	.	.	.	.	.	.	.	.	.	R	1741	167	NS3
.	.	.	.	.	.	.	.	.	.	.	.	.	.	.	.	.	.	.	.	.	.	.	.	.	.	.	I	.	.	.	.	T	1846	272	NS3
.	.	.	.	.	.	S	.	.	.	.	.	.	.	.	.	.	.	.	.	.	.	.	.	.	.	.	.	.	.	.	.	N	1978	404	NS3
.	.	.	.	.	.	.	.	.	.	.	.	.	.	.	.	.	.	.	.	.	V	.	.	.	.	.	.	.	.	.	.	M	2036	462	NS3
R	.	.	.	.	.	.	.	.	.	.	.	.	.	.	.	.	.	.	.	.	.	.	.	.	.	.	.	.	.	.	.	K	2048	474	NS3
S	S	S	S	S	S	S	S	S	S	S	S	S	S	S	S	S	S	S	S	S	S	S	S	S	S	S	S	S	S	S	S	G	2195	3	NS4A
.	.	.	.	.	.	.	.	T	.	.	.	*	.	.	.	.	.	.	.	.	.	.	.	.	.	.	.	.	.	.	.	A	2201	9	NS4A
.	.	.	.	.	.	.	.	.	.	.	.	*	.	.	.	.	.	.	.	.	V	.	.	.	.	.	.	.	.	.	.	A	2207	15	NS4A
S	S	.	S	S	S	S	S	S	S	S	S	*	S	S	S	S	S	S	S	S	S	S	S	S	S	S	S	S	S	S	S	L	2210	18	NS4A
.	.	.	.	.	.	.	.	.	.	.	.	*	.	.	.	v	V	.	.	.	.	.	.	.	.	.	.	.	.	.	.	I	2233	41	NS4A
.	.	.	.	.	.	.	.	.	.	.	.	*	.	.	.	.	.	.	.	.	.	.	.	Q	.	.	.	.	.	.	.	K	2241	49	NS4A
.	.	.	E	E	E	E	.	.	.	.	.	*	.	.	.	.	.	.	.	.	.	.	.	.	.	.	.	.	.	.	.	G	2288	96	NS4A
.	.	.	.	.	.	.	.	.	.	.	.	*	.	.	.	.	.	S	S	S	S	S	S	S	S	S	S	S	S	S	S	P	2298	106	NS4A
.	.	.	.	.	.	.	.	.	.	.	.	*	.	.	.	.	.	.	.	.	.	.	.	I	.	.	.	.	.	.	.	T	2359	18	NS4B
V	V	V	V	V	V	V	V	V	V	V	V	*	V	V	V	V	V	V	V	V	V	V	V	V	V	V	V	V	V	V	V	A	2383	42	NS4B
.	.	.	.	.	.	.	.	.	.	.	.	*	.	.	.	.	.	V	.	.	.	.	.	.	.	.	.	.	.	.	.	A	2419	78	NS4B
.	.	.	.	.	.	.	.	.	.	.	.	*	.	.	.	.	.	.	.	.	.	.	.	.	.	.	.	.	.	R	.	K	2554	213	NS4B
.	.	.	.	.	.	.	.	.	.	.	.	.	.	.	V	.	.	.	.	.	.	.	.	.	.	.	.	.	.	.	.	I	2706	107	NS5
E	.	.	.	.	.	.	E	.	.	.	.	.	.	.	.	.	.	.	.	.	.	.	.	.	.	.	.	.	.	.	.	G	3032	433	NS5
.	.	.	.	.	.	.	V	V	V	V	.	.	.	.	.	.	.	.	.	.	.	.	.	.	.	.	.	.	.	.	.	I	3095	496	NS5
.	.	.	.	.	.	.	.	.	.	.	.	*	.	.	.	.	.	.	.	.	.	.	.	.	.	.	.	.	.	I	.	T	3200	601	NS5
.	.	.	.	.	.	.	.	.	.	.	I	.	.	.	.	.	.	.	.	.	.	.	.	.	.	.	.	.	.	.	.	V	3414	815	NS5
.	.	G	.	.	.	.	.	.	.	.	.	.	.	.	.	.	.	.	.	.	.	.	.	.	.	.	.	.	.	.	.	A	3415	816	NS5
.	K	.	.	.	.	.	.	.	.	.	.	.	.	.	.	.	.	.	.	.	.	.	.	.	.	.	.	.	.	.	.	N	3417	818	NS5
.	A	.	.	.	.	.	.	.	.	.	.	.	.	.	.	.	.	.	.	.	.	.	.	.	.	.	.	.	.	.	.	G	3420	821	NS5
.	.	.	.	.	.	.	.	.	.	.	*	.	.	.	.	.	.	.	.	I	.	.	.	.	.	.	.	.	.	.	.	T	3424	825	NS5
.	.	*	.	.	.	.	.	V	.	*	*	.	.	.	.	.	*	.	.	.	.	.	.	.	.	.	.	.	.	.	.	A	3430	831	NS5

A dot indicates no amino acid difference between strains. Asterisks indicate sequencing gaps where the amino acid identity at the position is unknown. Protein and position indicate the amino acid position based on the Beh355964 genome (GenBank accession number KM267635), while the polyprotein number indicates the amino acid position relative to the CbaA4-4005 polyprotein.

**Fig 4:**
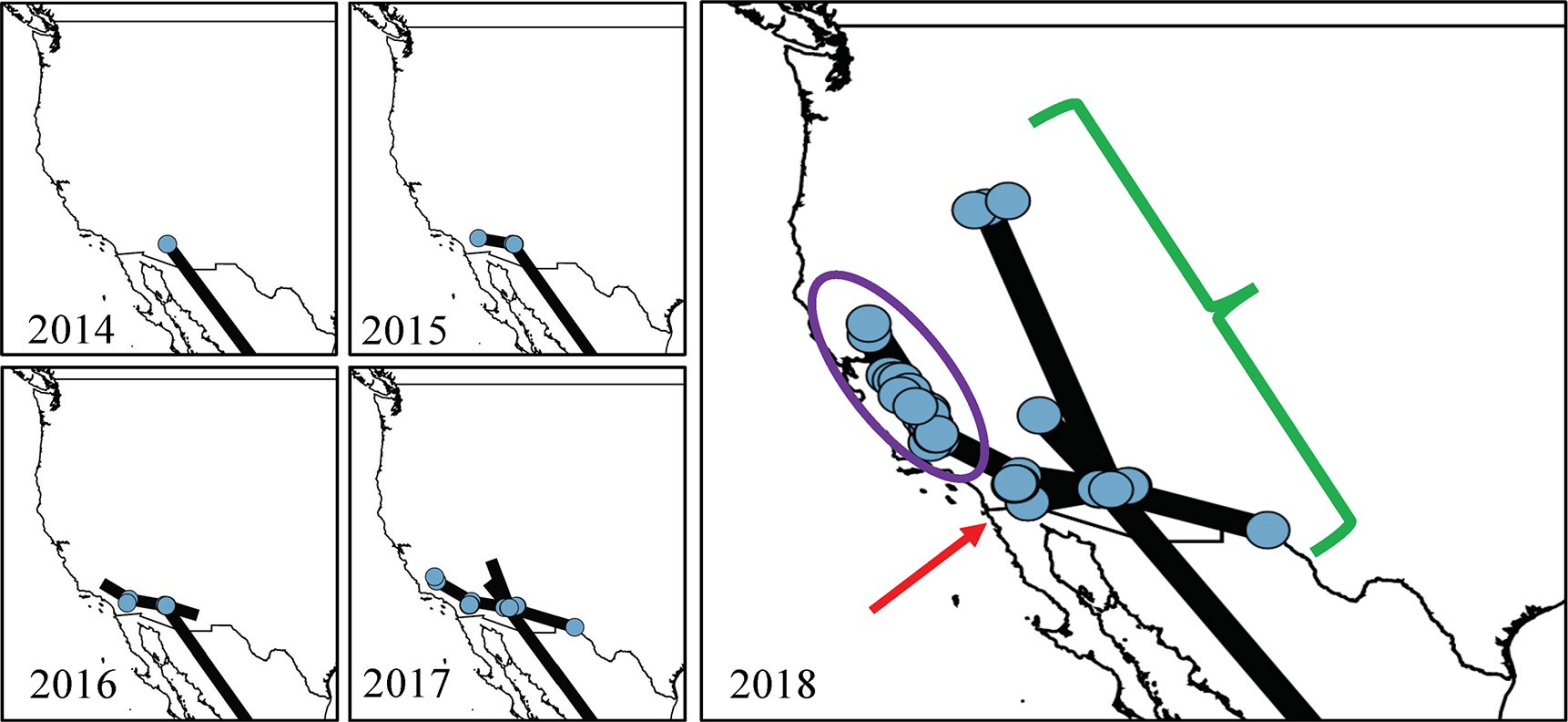
Geographic expansion of SLEV genotype III likely followed three routes throughout the Western US. Genomes and inferred ancestors are represented with blue circles. The inferred SLEV expansion is depicted for each year for 2014–2017 and a composite is shown for 2014–2018. Route one, shown by a red arrow, consists of SLEV expansion from AZ into Southern CA. Route two, circled in purple, involves SLEV transported from AZ into the Central Valley of CA. Route three, indicated with a green bracket, represents SLEV movement from AZ to all locations east of the Sierra Nevada mountains.

To define the geographic spread of SLEV in the western United States since 2014, a phylogeographic reconstruction was performed (Fig 4). All North American genotype III SLEV strains originated in AZ and three independent routes of SLEV expansion were identified. One route originated in AZ and expanded westward into Southern CA, and two routes projected northward on either side of the Sierra Nevada mountains.

## Discussion

SLEV is a re-emerging arthropod-borne virus that has caused significant outbreaks throughout the western US in recent years. To understand the recent re-emergence and spread of SLEV in the western US, 30 SLEV genomes were sequenced and characterized using phylogeographic approaches. All SLEV strains detected in the US prior to 2014 clustered in genotypes I, II or V [8,11,13], but all SLEV available for this study during and after 2014 clustered monophyletically in genotype III with the 1978 and 2005 Argentinian genomes. The most recent common ancestor of genotype III SLEV in the western US was estimated to have arisen around March of 2013, suggesting that all genotype III SLEV in the US is the result of a single introduction that occurred between August 2012 and October 2013. All three of the genotypes historically endemic to the US (genotypes I, II and V) were also identified in CA during 1955 and 1983, 1989, and 2003, respectively (Fig 2A). Due to the limited number of sequences available, it is unclear if these genomes circulated concurrently or sequentially in time.

Following the introduction of genotype III, SLEV has accrued multiple amino acid changes. Sixty-three amino acid substitutions were identified in genotype III SLEV genomes from the western US. Four amino acid substitutions were conserved among almost all genotype III sequences from the western US and five were conserved along internal phylogenetic branches. Additional studies are needed to determine if these substitutions arose stochastically or in response to local selection pressures.

The SLEV sequences from the western US studied here form four geographically distinct clusters, IIIa-d (Fig 3), in genotype III, and appear to have traveled along three independent routes (Fig 4). Support for three of the clades, IIIa, IIIc, and IIId, was very strong (posterior ~ 1); however, support for cluster IIIb was much weaker (posterior <0.2) and should be interpreted with caution. Further studies with additional genomes may help to clarify the relationships within the clusters.

The pattern of SLEV expansion suggests that mountain ranges; specifically, the Sierra Nevada, Cascade, and the Transverse Ranges (Fig 1B) have acted as natural barriers to the geographic expansion of SLEV in the Western US. This observation is consistent with limited evidence that suggests that WNV, which cycles in a bird-mosquito-bird pattern as well, has also been geographically restricted in a similar way [45,46]. While both WNV and SLEV are maintained in passerine birds throughout the US, WNV infects a wider range of bird species and causes more significant disease. Despite the broader host range, phylogenetic studies have demonstrated that WNV sequences from CA cluster together, suggesting a limited number of introductions into or out of the state (45, 46). Similar studies have also found that the westward expansion of WNV appears to have stalled upon reaching the Rocky Mountains (45). Together, these studies suggest that mountain ranges likely inhibit arbovirus expansion in general by influencing movement of vector mosquito and reservoir bird species. Elevation gradients (Fig 1B) are associated with rapid changes in temperature (Fig 1C), vegetation, land use and precipitation, all of which significantly impact and often restrict the distribution of mosquito and bird populations.

In the western US, the Tehachapi Mountains in the Transverse Range [47,48] as well as the Cascade [47–49] and Sierra Nevada ranges [47–49] limit gene flow among populations of *Cx*. *tarsalis*. In southeastern CA, *Cx*. *tarsalis* abundance is inversely related to elevation [50]. Genetic structure has also been reported within the *Cx*. *pipiens* complex in the western US. *Cx*. *quinquefasciatus* are restricted south of the Tehachapi Mountains, *Cx*. *pipiens* are found in northern CA (north of 39°N) (Fig 1A), and a hybrid zone of the two species is found in the Central Valley of CA [51–55]. Given that *Cx*. *pipiens quinquefasciatus* hybrids are more efficient transmitters of the closely related WNV than either *Cx*. *pipiens* or *Cx*. *quinquefasciatus* [56], it is possible that variation in vector competence among mosquitoes in the *Cx*. *pipiens* complex has also influenced the expansion of SLEV, especially because no SLEV-positive mosquito pools have been detected in CA north of the 40°N (Fig 1A) [22] where *Cx*. *pipiens* are most prevalent.

The relationship between mountain ranges and SLEV dispersal is further complicated by involvement of the avian hosts, passeriform and columbiform birds. While birds are more mobile than mosquitoes, there is evidence that the Sierra Nevada, Cascade, and Transverse Mountain Ranges are also barriers for gene flow within some passerine bird species [57–60], where mountains restrict bird dispersal within breeding grounds. Mountain ranges influence the migration of passerine birds in the western US as elevation gradients drive seasonal fluctuations in ecological productivity [61]. Considering the well-documented effect of elevation gradients on gene flow and migration of Passeriform birds, as well as the geographic variation in the abundance of SLEV susceptible mosquitoes, reduced SLEV dispersal across mountain ranges, deserts, or other environments unsuitable for reservoir hosts or vectors can be expected [51–55,62].

In addition, the Mojave and Colorado deserts in the southwestern US may have also contributed to the pattern of SLEV expansion. Mosquito populations relay on the maintenance of aqueous larval habitats originating from natural (winter rainfall, expansion of salt marshes along the Salton Sea, CA) and anthropogenic (residential and agricultural irrigation) water sources [50,62,63]. The extreme arid conditions of the Mojave and Colorado deserts results in an irregular distribution of mosquito larval habitats with some areas, such as irrigated valleys and the Salton Sea, supporting large mosquito populations, while other areas support very few. The heterogenous distribution of mosquitoes in the southwestern US may have further restricted the spread of SLEV.

Given the diverse ecologies of the western US, variation in bird and mosquito species distributions may influence viral persistence and dissemination patterns. In central CA and AZ, a single introduction of SLEV was maintained locally year-to-year, while multiple short-lived introductions were observed in southeastern CA. The ecologies of southern AZ and southeastern CA are more similar, as both are arid deserts dotted with cities and smaller towns. Whether SLEV persists in a particular area may be attributable in part to the patchiness of the environment. As for Eastern equine encephalitis virus, another bird-transmitted arbovirus [64], our findings suggest source-sink dynamics in which larger areas with interconnected patches of suitable host and vector habitat maintain larger and more robust viral metapopulations, while smaller more isolated ecological “islands” (sinks) in the middle of deserts could leave SLEV more vulnerable to stochastic fadeout. Maricopa County, AZ is home to Phoenix, which is surrounded by several irrigated valleys, collectively make for a large area suitable for the hosts and vectors of SLEV. Southeastern CA has smaller urban areas from Palm Springs to Indio and a series of smaller towns on both ends of the Salton Sea in the irrigated Coachella and Imperial Valleys. Other unidentified differences in the local micro-environments or anthropogenic factors, such as vector management strategies or water use, may have contributed to the differences observed between the persistence of SLEV in AZ and the rapid extinction of SLEV lineages introduced into southeastern CA. It is also possible that year-to year persistence occurred in southern CA but was not detected by our study because sequences were available only from a few well-sampled areas of the desert.

Finally, while the natural dispersal of SLEV-competent birds and mosquitoes seem like the most obvious drivers of SLEV expansion, anthropogenic transport should not be neglected. While humans are considered dead-end hosts for SLEV and cannot perpetuate the SLEV transmission cycle, human behavior could have facilitated the transport of SLEV-infected mosquitoes. Transport and survival of mosquitoes has been reported in personal automobiles [65], as well as during international flights aboard aircraft [35]. Human travel may facilitate the dispersal of SLEV beyond the ecological barriers that influence host and vector movement. For instance, range expansion of SLEV in the Central Valley of CA, an important agricultural region, may have been accelerated by infected mosquitoes hitchhiking in vehicles carrying crops.

In this study, we failed to detect any historically endemic SLEV genotypes (genotypes I, II or V) in the western US after 2014, suggesting that all SLEV activity in the western US is the result of the recent introduction of genotype III. However, it is unclear if historically endemic SLEV genotypes continue to circulate in the remaining portions of the US. Our results also revealed three distinct routes of SLEV dissemination that support the hypothesis that geographic and ecological features, likely the Sierra Nevada, Cascade, and the Transverse Ranges, influenced the movement of SLEV in the western US. It is possible that similar geographic barriers may also influence the movement of other avian arboviruses in the US, like WNV and Western equine encephalitis virus, the latter of which has not been detected in CA since 2006, but which re-emerged in Mexico in 2019 [66]. Understanding natural barriers to virus dissemination may allow public health officials to exploit geographic features affecting arbovirus spread to better protect local communities and to tailor mitigation strategies to areas that are more susceptible to virus migration, such as low-elevation valleys. Taken together, the results of this study highlight the importance of viral genome sequencing in virus surveillance.
